# Bloody Perspiration

**Published:** 1831

**Authors:** 


### XXVI.

### Bloody Perspiration.

A curious case of this kind is related in the French "Transactions Medicales" for November last.

*Case.* A young woman, aged twenty-one years, irregular in menstruation, and of indolent habits and obstinate temper, had been much irritated by some reflections made by her parents, on account of her abjuring the Protestant religion. She left her paternal roof, and after wandering about for some time, took up her residence in an hospital. She then was suffering violent attacks of hysteria, attended with general convulsions, and exquisite sensibility in the pubic and hypogastric regions. After paroxysms of hysteria, which sometimes lasted 24 or 36 hours, this female fell into a kind of ecstacy, in which she lay with her eyes fixed, sensibility and motion suspended. Sometimes she muttered a prayer; but the most remarkable phenomenon was an exudation of blood from the cheeks and the epigastrium, in the form of perspiration. The blood exuded in drops, and tinged the linen. The cutaneous surface appeared injected in those parts whence the blood escaped, being red, and shewing a net-work of arborescent vessels. This bloody perspiration took place every time that the hysteric paroxysm continued for a considerable time. This state continued for three months; and ultimately gave way, it is, said, to local bleeding about the head and sexual organs, together with strong revulsive measures.

---

